# Digitale Gesundheitsanwendungen bei Borderline-Persönlichkeitsstörung

**DOI:** 10.1007/s00115-025-01856-0

**Published:** 2025-07-22

**Authors:** Gitta Jacob, Eva Fassbinder, Jan Philipp Klein

**Affiliations:** 1 GAIA, Hans-Henny-Jahnn-Weg 53, 22085 Hamburg, Deutschland; 2 Zentrum für Integrative Psychiatrie Kiel, Klinik für Psychiatrie und Psychotherapie, Christian-Albrechts-Universität zu Kiel, Kiel, Deutschland; 3 Privatpraxis für Psychische Gesundheit, Lübeck, Deutschland; 4 Kliniken für Psychiatrie, Psychosomatik und Psychotherapie, Universität zu Lübeck, Lübeck, Deutschland

**Keywords:** Therapeutische Beziehung, Kristenintervention, Schematherapie, Skills-Training, Sicherheit, Therapeutic relationship, Crisis intervention, Schema therapy, Skills training, Safety

## Abstract

Digitale Gesundheitsanwendungen (DiGAs) bei der Borderline-Persönlichkeitsstörung (BPS) sind noch ein neues und im Verhältnis wenig erforschtes Thema, allerdings mit großem Potenzial gerade in Anbetracht der Tatsache, dass es für BPS kein einziges zugelassenes Medikament gibt. Bisher ist eine DiGA für BPS (priovi) vom Bundesinstitut für Arzneimittel und Medizinprodukte (BfArM) dauerhaft zugelassen. Bedenken bei diesem Thema betreffen die Sicherheit der Patientinnen und das Fehlen der Therapiebeziehung – allerdings fanden sich in Studien zu priovi im Gegenteil positive Befunde zur therapeutischen Beziehung und die Sicherheit scheint sich dadurch eher zu erhöhen. Generell ist in diesem Bereich jedoch noch viel Forschungsbedarf vorhanden. In der konkreten Anwendung muss jeder Behandler die individuelle Situation der Patienten berücksichtigen und eine DiGA so in den Behandlungsplan integrieren, wie es für diese Patienten passend erscheint.

**Zusatzmaterial online:**

Die Online-Version dieses Beitrags (10.1007/s00115-025-01856-0) enthält eine Übersicht über die Begriffsdefinitionen.

## Abstract (en)

Digital health applications (DiGA) for borderline personality disorder (BPD) are still a relatively new and underresearched topic but hold great potential, especially considering that there is no single approved medication for BPD. So far, one DiGA (priovi) has been permanently approved for BPD by the Federal Institute for Drugs and Medical Devices (BfArM). Concerns related to this topic mainly relate to patient safety and the lack of a therapeutic relationship; however, studies on priovi have actually shown positive findings regarding the therapeutic relationship and its use seems to increase safety rather than compromise it. That said, there is still a significant need for further research in this field. In the practical application each clinician must consider the individual situation of their patient and integrate a DiGA into the treatment plan in a way that seems to best suit the specific patient’s needs.

Digitale Gesundheitsanwendungen (DiGAs) werden bisher vor allem bei häufigen Indikationen wie Schlafstörungen, Depressionen und Angststörungen eingesetzt. Hier gilt die Behandlung als klar strukturierbar und die Patienten als gut führbar, solange keine akute Suizidalität besteht. „Schritt-für-Schritt-Manuale“ sind in der Psychotherapie etabliert und lassen sich digital überzeugend umsetzen. Die Wirksamkeit digitaler Anwendungen wurde bei vielen dieser Indikationen gezeigt [1]. Die Borderline-Persönlichkeitsstörung (BPS) gilt hier aufgrund der damit verbundenen Krisen, wechselnden Themen und größeren Komplexität von Manualen als anspruchsvoller. Dennoch zeigen DiGAs auch hier zunehmend ihren Wert.

## DiGAs für BPS: Chancen und Risiken

Aus vielen Jahren Forschung und Versorgung im BPS-Bereich kennen wir im Zusammenhang mit DiGAs in dieser Gruppe einige zentrale Bedenken, die wir selbst zu Beginn unserer Arbeit in diesem Bereich durchaus teilten.

- BPS-Patienten benötigen viel menschliche Ansprache, die therapeutische Beziehung stellt einen wichtigen Grundpfeiler der Behandlung dar und ohne diese ist eine Psychotherapie kaum möglich.
- Die schnell wechselnden Themen und häufigen Krisen in dieser Patientengruppe erfordern eine thematische Flexibilität, die von einer DiGA kaum geleistet werden kann.
- Suizidalität und gefährliches Verhalten sind prominent und können nicht automatisiert oder digitalisiert abgefangen werden – das Risiko dafür kann durch digitale Therapien möglicherweise noch gesteigert werden.

Auf der anderen Seite wird hier schon lange viel Potenzial gesehen, denn viele BPS-Patienten sind falsch und unterversorgt und aufgrund von Fachkräftemangel und generellen Engpässen in der Versorgung ist hier auch keine Besserung in Sicht.

- Es gibt mittlerweile mehrere störungsspezifische Psychotherapien für BPS mit nachgewiesener Evidenz [2]. Diese sind in Manualen beschrieben und zumindest in Teilen so strukturiert, dass eine Digitalisierung möglich erscheint.
- Nur wenige Psychotherapeutinnen sind für diese Patientengruppe ausreichend weitergebildet. Gerade in ländlichen Gebieten ist spezifische Therapie praktisch nicht zeitnah zu bekommen [3]. Hier tut Abhilfe dringend not.
- In Deutschland wird aufgrund der Mangelsituation bei BPS-Patienten häufig auf stationäre Psychotherapieangebote ausgewichen. Diese sind jedoch nicht wirksamer als ambulante Versorgung und gehen mit massiven Nachteilen einher: enorm höhere Kosten, Gefahr der Hospitalisierungsförderung und mangelnde Integration des Gelernten in den Alltag, vor allem wenn die Klinik weit vom Heimatort entfernt ist.
- Für die BPS ist kein einziges Medikament zugelassen – im Gegenteil warnen die Leitlinien sogar vor Übermedikation [4]. Dadurch stehen Betroffene im persönlichen Behandlungssystem letztlich oft ohne jede adäquate Behandlung dar.

## Stand der Forschung

Trotz des großen Potenzials und Interesses lassen sich bisher relativ wenige Entwicklungen digitaler Angebote finden, die dem Anspruch an eine DiGA gerecht werden könnten. Anwendungen unter dem Schlagwort „internet-delivered treatment“ beziehen sich fast ausschließlich auf die Verlegung von persönlichen Therapien in ein Video- oder E‑Mail-Setting [5–7]. In einer 2024 erschienenen Metaanalyse wurden 40 Publikationen zu 38 Interventionen mit insgesamt 6611 Teilnehmenden zusammengefasst [9]. Dabei fokussierten die meisten Anwendungen jedoch nur auf einzelne BPS-Symptome (z. B. Suizidgedanken, Paranoia, Selbstverletzungen) oder nur einen oder wenige Skills; zudem wurden auch nicht nur Borderline-Patienten eingeschlossen, sondern offenbar generell Studien zu DBT („dialectical behavioral therapy“) mit einbezogen. Zum Beispiel wird von Wilks und Kollegen eine Online-DBT-Anwendung speziell nur für die Reduktion von Suizidgedanken und exzessivem Alkoholkonsum getestet und hier nur ein Effekt auf die Alkoholexzesse gefunden [10]. Insgesamt zeigte sich metaanalytisch kein signifikanter Effekt auf BPS-Symptome oder allgemeine Psychopathologie, sondern nur auf die Einzelsymptome „Suizidgedanken“ und „Paranoia“.

Selbst neuere Arbeiten stellen eher kleine Pilotstudien zu sehr begrenzten Programmen dar (z. B. [11]). Auch ein recht aktuelles systematisches Review von 2022 kommt zu dem Schluss, dass Digitalisierung in der Therapie von BPS ein großes Potenzial besitzt und sich erste Hinweise auf Wirksamkeit zeigen, das aber noch von viel zu wenig Forschung gestützt wird [12]. Eine relativ frühe Ausnahme war eine 2017 veröffentlichte randomisierte kontrollierte Studie (RCT) aus der Arbeitsgruppe von Mary Zanarini, in der positive Effekte eines psychoedukativen Onlineprogramms bei BPS gefunden wurden [13].

Vor diesem Hintergrund stellen unsere Arbeiten zu den beiden Programmen priovi® und alivis® (beide entwickelt und betrieben von GAIA, Hamburg) nach unserer Kenntnis aktuell die einzigen Projekte dar, in denen umfangreiche Onlinetherapien entwickelt und getestet werden bzw. wurden, die eine Reduktion der gesamten BPS-Symptomatik und nicht nur einzelner Symptome anstreben. Zudem ist priovi die einzige bislang dauerhaft zugelassene DiGA mit der Indikation BPS, und für alivis wird eine solche Zulassung angestrebt. Daher erscheint eine genauere Darstellung sowohl der Programme als auch der dazu bislang vorliegenden Befunde im Rahmen dieses Themenheftes angebracht.

## priovi® – die aktuell einzige DiGA für BPS

Die Selbstmanagementintervention (SMI) priovi, deren Entwicklung 2013 startete und die 2023 als erste und bis zur Abgabe des Manuskriptes einzige DiGA für BPS zunächst vorläufig, 2024 dann auch dauerhaft zugelassen wurde, orientiert sich an der Schematherapie. In diesem Ansatz werden die Symptome, Probleme und Ressourcen der Patientin in einem sog. Modusmodell abgebildet und behandelt. Für ein Onlineprogramm bietet sich dieses Konzept an, da es einerseits hoch strukturiert ist und etwa die Bearbeitung bestimmter Themen in einer bestimmten Reihenfolge nahelegt. Gleichzeitig ist es breit angelegt – prinzipiell können sehr viele Probleme und Symptome damit verstanden und behandelt werden.

**Abb. 1:**
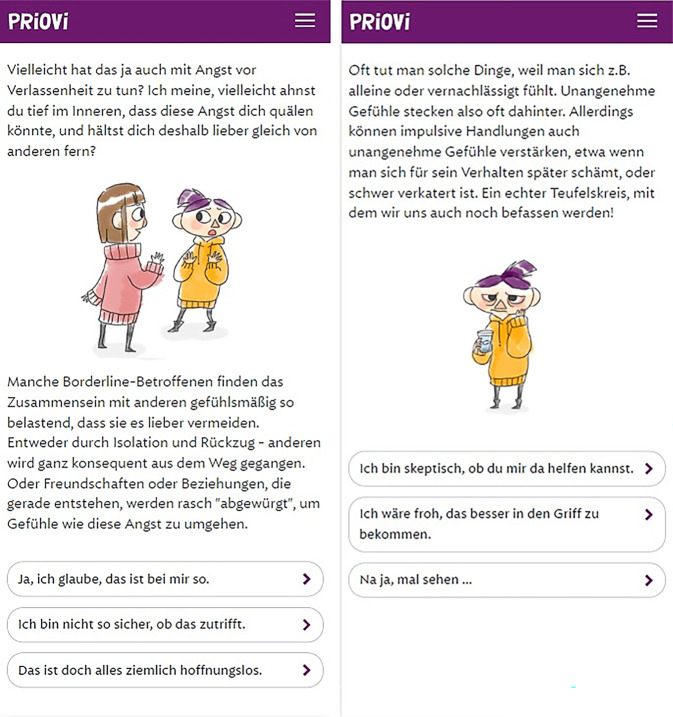
priovi-Screenshots

In priovi startet die Nutzerin zunächst mit einer umfangreichen Psychoedukation zur BPS-Diagnose, dem biografischen Hintergrund ihrer Probleme und der Schematherapie. Im Anschluss wird mit der Patientin ein individuelles Modusmodell erarbeitet. Dem folgt ein umfangreiches Angebot von Übungen für die verschiedenen Modi. Der Umfang variiert in Abhängigkeit von den Themen und Problemen der jeweiligen Nutzerin; er entspricht jedoch etwa einem halben Jahr regelmäßiger Psychotherapie (Abb. 1).

Die Umsetzung erfolgt in einem simulierten Dialog, in dem die Nutzerin auf Informationen des Programms unterschiedliche Antwortmöglichkeiten hat; entsprechend ihrer Antworten passt sich das Programm an. Zusätzlich beinhaltet priovi Elemente wie Arbeitsblätter, Übungen, regelmäßige Nachrichten oder ein Symptomtracking. Die Ansprache ist ausgerichtet am schematherapeutischen Beziehungsstil des „limited reparenting“, liebevoll und warmherzig.

Aufgrund der oben genannten Befürchtungen im Zusammenhang mit Onlinetherapie für BPS wurde priovi in der Entwicklung intensiv qualitativ begleitet, um zunächst „usability“ und Sicherheit zu überprüfen. Eine erste Pilotstudie zeigte hier gute Ergebnisse [14]. Zudem wurde die Frage, inwieweit die fehlende menschlich-therapeutische Beziehung für die Nutzung eines Onlineprogramms schwierig ist, in einer qualitativen Studie untersucht [15]. Dabei zeigte sich, dass die Patientinnen durchaus auch zu priovi eine sehr positive therapeutische Beziehung aufbauen. Dabei fehlen manche Qualitäten der menschlichen Beziehung – etwa, dass das aktuelle Tagesgeschehen nicht besprochen werden kann. Auf der anderen Seite zeigt die Beziehung zu priovi auch Merkmale, die es Patientinnen leichter machen, sich mit den therapeutischen Inhalten auseinanderzusetzen. Dazu gehört insbesondere, dass man nicht befürchten muss, von priovi negativ bewertet zu werden.

In einer ersten RCT wurden nur Patienten mit BPS rekrutiert, die in psychiatrischer oder psychotherapeutischer Behandlung waren und deren Psychiater/Therapeut der Studienteilnahme zustimmte. Hier zeigte sich eine sehr gute Akzeptanz, hohe Sicherheit und – statistisch signifikant allerdings nur in der Per-Protokoll-Analyse (d. h. bei Patientinnen, die priovi mehr als 3 h genutzt haben) – eine Tendenz zu positiver Wirksamkeit von priovi auf die BPS-Symptomatik gemessen mit der Borderline-Symptomliste(BSL)-23 [16].

Daher wurde nach einer Überarbeitung von priovi eine weitere RCT durchgeführt, in dem nun auch Patienten mit BPS teilnehmen konnten, die aktuell nicht in Behandlung waren – in Anbetracht der Versorgungslage ist diese Zielgruppe besonders wichtig. Hier zeigte sich eine signifikante Überlegenheit von priovi im Vergleich zur Kontrollgruppe in der BSL-23 [17]. Auch in den sekundären Endpunkten „depressive Symptomatik“ und „Angstsymptomatik“ (Generalized Anxiety Disorder 7‑Item Scale, GAD-7) verbesserten sich priovi-Patientinnen signifikant mehr als die Kontrollgruppe. Zudem wurde die Verringerung der Anzahl der Suizidversuche signifikant in dem Sinne, dass Nutzerinnen von priovi im Follow-up weniger Suizidversuche berichteten als die Kontrollgruppe. Die Nutzerinnen von priovi waren hoch zufrieden mit dem Programm. Diese positiven Ergebnisse führten auch zur dauerhaften Aufnahme ins DiGA-Verzeichnis des BfArM.

## alivis® – eine Synthese der „3. Welle“ für BPS

Die Onlinetherapie alivis wurde zwischen 2021 und 2023 klinisch entwickelt. Sie folgt technisch dem gleichen Dialogprinzip wie priovi und enthält ebenfalls einen umfangreichen Dialog sowie viele weitere Elemente. Konzeptuell stellt sie eine Synthese verschiedener „3.-Welle-Ansätze“ für BPS dar und integriert DBT, die den Schwerpunkt der Anwendung darstellt, mit Akzeptanz-und-Commitment-Therapie (ACT), Self-Compassion-Therapy und Cognitive Behavioral Analysis System of Psychotherapy (CBASP). Dabei wird natürlich von allen Ansätzen der Fokus auf BPS-typische Probleme und Symptome gelegt.

Die Integration von ACT-Techniken erfolgte, da die Distanzierung von schwierigen Gefühlen und Gedanken für viele BPS-Patienten außerordentlich wichtig ist und das ACT-Konzept dies noch einmal umfassender und mit Bezug zu den eigenen Werten beschreibt als dies im Skills-Ansatz der DBT erfolgt. Die Ergänzung um Werteorientierung ergibt sich aus der häufigen Rückmeldung von Patientinnen, dass skillorientierte Behandlungen manchmal zu kurz greifen und dass die Orientierung auf eigene Werte in besonderem Maße dazu motiviert, gesünderes Verhalten zu lernen und einzuüben. Der Kiesler-Kreis wurde aufgenommen, da viele BPS-Patientinnen dazu neigen, sich submissiv-feindselig zu anderen Menschen in Bezug zu setzen und damit auf ähnliche Probleme stoßen wie chronisch depressive Patienten.

### Infobox 1 Inhalte von alivis

- Psychoedukation zur Borderline-Persönlichkeitsstörung (BPS) und Behandlungstechniken
- DBT(„dialectical behavioral therapy“)-Stresstoleranzskills und Techniken gegen Dissoziation
- Werteorientierte Aktivierung und Ausrichtung im Leben
- Aufbau von Verständnis für, Umgang mit und Regulation eigener Emotionen
- Umgang mit belastenden Gedanken (Vermittlung von Defusionstechniken)
- Selbstmitgefühl
- Soziale Fertigkeiten und Umgang mit Beziehungen unter Einbezug des Kiesler-Kreises

**Abb. 2:**
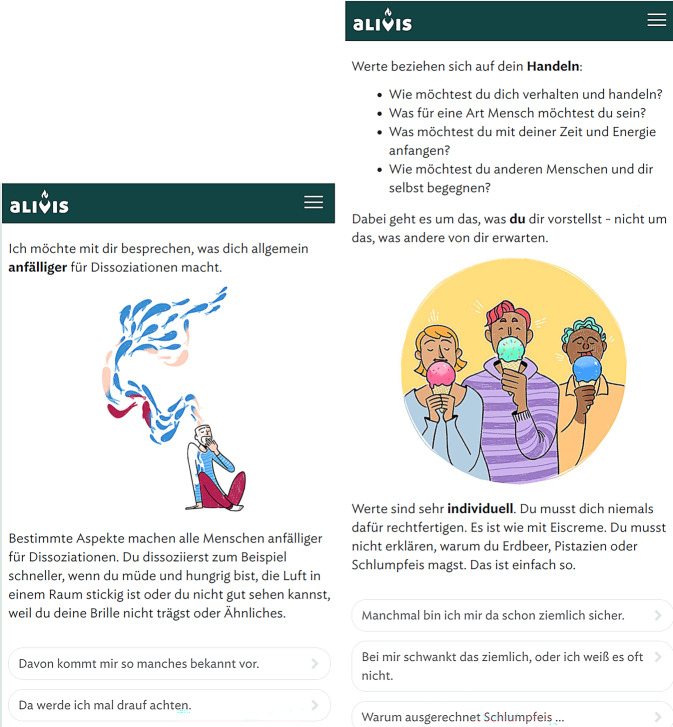
alivis-Screenshots

Im Unterschied zu priovi beinhaltet alivis auch einen Dialog zum Umgang mit akuten Krisen. Die Nutzerin kann zu Beginn einer „Sitzung“ mit alivis angeben, ob sie regulär mit dem therapeutischen „Dialog“ fortfahren möchte oder ob sie sich in einer akuten Krise befindet, die akut gelöst werden muss. In diesem Krisendialog übt sie dann ein, Abstand zur aktuellen Situation zu gewinnen und geeignete Skills einzusetzen, um sich wieder zu beruhigen. alivis wurde wie priovi unter enger Beteiligung von Patientinnen entwickelt und schon frühzeitig Feedback eingeholt und das Programm entsprechend überarbeitet. Mittlerweile wurde das fertige Produkt von etwa 40 Personen mit BPS genutzt. Die ersten Erfahrungen sind sehr positiv, sprechen für eine mindestens so gute Wirksamkeit wie priovi und insbesondere auch eine gute Akzeptanz des „Krisendialogs“. Eine RCT zur Testung der Wirksamkeit im Vergleich zu „treatment as usual“ hat Ende 2024 mit der Rekrutierung gestartet (Registrierungsnummer NCT06601907 in clinicaltrials.cov; Abb. 2).

## Umgang mit Krisen und Notfällen bei priovi und alivis

Sobald es um Onlineangebote für BPS-Patienten geht, stellt sich die Frage nach dem Umgang mit Krisen und Notfällen. Ein klares und sicheres Konzept ist hier zwingend notwendig. Gleichzeitig ist es immer wichtig, zu beachten, dass ein digitales Angebot genau wie jede andere therapeutische oder psychiatrische Maßnahme immer nur ein Element des Behandlungsplanes darstellt. Es liegt im Ermessen der behandelnden Person, einzuschätzen, welchen Stellenwert das bei einem gegebenen Patienten haben kann oder soll. Bei stark krisenhaften Fällen, die häufig eskalieren, kann ein Onlineangebot natürlich nicht das ganze Behandlungssystem ersetzen und eine vollständige Behandlung jedes Notfalls gewährleisten. Sie muss jedoch Werkzeuge beinhalten, um dem Nutzer zunächst bei der eigenständigen Krisenbewältigung zu helfen, und im Falle eskalierender Situationen in den richtigen Momenten auch ihre Grenzen aufzeigen und Hinweise zum Aufsuchen intensiverer Hilfen geben. Zudem muss sie so gestaltet sein, dass ihre Nutzung nicht ihrerseits bereits zu Krisen und Eskalationen führt.

### Infobox 2 Elemente zur Bewältigung von Notfällen und Krisen in priovi und alivis

- Inhärent sicheres Design: Nutzer können jederzeit emotionalen Stress adressieren, schwierige Themen auslassen und Techniken zur Beruhigung nutzen. Beispielsweise werden häufig Antwortalternativen angeboten, mit denen Nutzer ausdrücken können, dass sie sich akut belastet fühlen, und dann werden Techniken zur Beruhigung angeboten.
- Positive „Reparenting“-Beziehungsgestaltung, die Gefühle von Sicherheit und Entspannung fördert und deeskalierend wirkt.
- Übungen zur Entspannung und zum Aufbau von Sicherheit und Gelassenheit.
- Erstellen eines Notfallplans, um im Fall von Krisen alle wichtigen akuten Maßnahmen und Kontakte zur Hand zu haben (nur in alivis).
- Angebot eines Krisengespräches, das als erste Maßnahme in einer Krise genutzt werden kann (nur in alivis). In diesem Modul wird analog zum Umgang mit Krisen in der DBT („dialectical behavioral therapy“) umgegangen. Zunächst wird die aktuelle Anspannung erfragt und darauf dann differenziell reagiert. Bei sehr hoher Anspannung werden Stresstoleranzskills vorgeschlagen; sofern die Nutzerin bereits eine Skillskette erstellt hat, wird diese eingesetzt. Bei etwas niedrigerer Anspannung, oder wenn sich die Anspannung durch die Stresstoleranzskills etwa gelegt hat, werden Skills zur Emotionsregulation angeboten und abschließend eine Handlung in Richtung der oder gegen die aktuelle Emotion geplant.

## Bewertung

Digitale Gesundheitsanwendungen haben ein großes Potenzial für die Behandlung der BPS. Bislang ist nur priovi vom BfArM dauerhaft zugelassen. Da es kein einziges zugelassenes Medikament gegen die BPS gibt, handelt es sich dabei somit um das – übrigens weltweit – erste verschreibungsfähige Therapeutikum bei dieser Störung. Die größten Bedenken, nämlich hinsichtlich Sicherheit, Suizidalität und die Verschlimmerung von Krisen, konnten durch umfangreiche qualitative und quantitative Studien ausgeräumt werden. Die Verbesserung hinsichtlich der Anzahl der Suizidversuche unter priovi spricht sogar eher dafür, dass das Programm die Sicherheit erhöhen kann. Auch das befürchtete Problem, dass den Nutzerinnen von Onlineprogrammen die menschliche Beziehung zu sehr fehlt, konnte ausgeräumt werden.

## Einsatz- und Verschreibungsmöglichkeiten

Digitale Gesundheitsanwendungen sind ein Element in der Behandlung psychischer Erkrankungen, die alle niedergelassenen Ärzte und Psychotherapeutinnen mit KV(Krankenversicherung)-Zulassung ihren GKV(gesetzliche Krankenversicherung)-Patienten verschreiben können. Im Rahmen des Entlassmanagements ist eine Verschreibung auch im stationären Kontext möglich. PKV(private Krankenversicherung)-Patienten müssen eine Erstattung mit ihrer Krankenkasse absprechen, die in vielen Fällen zumindest teilweise erfolgt.

Viele Kollegen empfehlen eine Verschreibung entweder zu Beginn einer Behandlung, zur Überbrückung einer Wartezeit oder zur Intensivierung einer laufenden Therapie. Allerdings spricht prinzipiell nichts dagegen, eine DiGA auch im späteren Verlauf der Behandlung oder auch am Ende der Behandlung einzusetzen, um der Patientin noch etwas Unterstützung mitzugeben. Letztlich entscheidet immer die verschreibende Person über den Einsatz und muss dafür abwägen, was für diesen Patienten hilfreich erscheint.

## Fazit für die Praxis

- Bei der Borderline-Persönlichkeitsstörung (BPS) sind digitale Gesundheitsanwendungen (DiGAs) noch relatives Neuland, auch wenn hier sicherlich ein großes Potenzial besteht. Wichtige Herausforderungen sind die Komplexität der Störung sowie der Umfang mit Krisen und Notfällen. priovi ist bisher das einzige Therapeutikum für BPS, das als DiGA verschrieben werden kann.
- Onlinetherapien müssen – wie ein Medikament – immer in den Behandlungsplan passen und mit der Patientin besprochen werden. Eine DiGA ist lediglich ein Werkzeug in therapeutischen Koffer des Behandlers. Sie kann ein „erster Schritt“ im Therapieprogramm sein, als Ergänzung zur Einzel- mit Gruppentherapien eingesetzt werden oder für bestimmte Patienten völlig ungeeignet sein.
- Im Bereich der Forschung sind insbesondere in der Indikation BPS noch viele Fragen offen – etwa die Frage der differenziellen Indikation. Generell gibt es noch wenige Studien zu umfangreicheren Ansätzen und zur Reduktion der gesamten Borderline-Symptomschwere durch digitale Ansätze.

## Supplementary Information

Wichtige Begriffe in der digitalen Gesundheitsversorgung im Bereich Psychiatrie und Psychotherapie
