# Erratum zu: Digitale Gesundheitsanwendungen bei Borderline-Persönlichkeitsstörung

**DOI:** 10.1007/s00115-025-01931-6

**Published:** 2025-12-19

**Authors:** Gitta Jacob, Eva Fassbinder, Jan Philipp Klein

**Affiliations:** 1 GAIA, Hans-Henny-Jahnn-Weg 53, 22085 Hamburg, Deutschland; 2 Zentrum für Integrative Psychiatrie Kiel, Klinik für Psychiatrie und Psychotherapie, Christian-Albrechts-Universität zu Kiel, Kiel, Deutschland; 3 Privatpraxis für Psychische Gesundheit, Lübeck, Deutschland; 4 Kliniken für Psychiatrie, Psychosomatik und Psychotherapie, Universität zu Lübeck, Lübeck, Deutschland

**Erratum zu:**

**Nervenarzt 2025**

10.1007/s00115-025-01856-0

Der Artikel „Digitale Gesundheitsanwendungen bei Borderline-Persönlichkeitsstörung“, geschrieben von Gitta Jacob, Eva Fassbinder und Jan Philipp Klein wurde ursprünglich unter exklusiver Lizenz von „© The Author(s), under exclusive licence to Springer Medizin Verlag GmbH, ein Teil von Springer Nature 2025“ veröffentlicht. Als Ergebnis der anschließenden Entscheidung den Artikel im Open-AccessModell zu veröffentlichen, wurde der Copyright-Vermerk des Artikels am 18. November 2025 in „© The Author(s) 2025“ geändert.

Dieser Artikel wird unter der Creative Commons Namensnennung 4.0 International Lizenz veröffentlicht, welche die Nutzung, Vervielfältigung, Bearbeitung, Verbreitung und Wiedergabe in jeglichem Medium und Format erlaubt, sofern Sie den/die ursprünglichen Autor(en) und die Quelle ordnungsgemäß nennen, einen Link zur Creative Commons Lizenz beifügen und angeben, ob Änderungen vorgenommen wurden.

Die in diesem Artikel enthaltenen Bilder und sonstiges Drittmaterial unterliegen ebenfalls der genannten Creative Commons Lizenz, sofern sich aus der Abbildungslegende nichts anderes ergibt. Sofern das betreffende Material nicht unter der genannten Creative Commons Lizenz steht und die betreffende Handlung nicht nach gesetzlichen Vorschriften erlaubt ist, ist für die oben aufgeführten Weiterverwendungen des Materials die Einwilligung des jeweiligen Rechteinhabers einzuholen.

Weitere Details zur Lizenz entnehmen Sie bitte der Lizenzinformation auf http://creativecommons.org/licenses/by/4.0/deed.de.

