# Supplementary material for: Digital health applications in borderline personality disorder
Source: Nervenarzt. 2025 Jul 22;96(5):445–50. [Article in German] doi: 10.1007/s00115-025-01856-0 (PMC12717144; doi:10.1007/s00115-025-01856-0)
Supplement: Supplementary file 1 — Wichtige Begriffe in der digitalen Gesundheitsversorgung im Bereich Psychiatrie und Psychotherapie [file 115_2025_1856_MOESM1_ESM.pdf]

Infobox: Wichtige Begriffe in der digitalen Gesundheitsversorgung im Bereich Psychiatrie und Psychotherapie

J. Kaminski[1,2,3], Simon Huldreich Kohl,[4,5] F. Machleid[1,2], C. Wiegmann [1,2,6], J.P. Klein[7], O. Rakitzis[1], R. Haaf[1], S. Schreiter[1], M. Preiß[6,7], L. Pezawas[8,9], Ingrid Titzler[10]

1 Department of Psychiatry and Neurosciences, Charité Campus Mitte, Charité – Universitätsmedizin Berlin, Corporate Member of Freie Universität Berlin, Humboldt-Universität zu Berlin, and Berlin Institute of Health, Berlin, Germany
2 Berlin Institute of Health at Charité – Universitätsmedizin Berlin, Germany
3 Recovery Cat GmbH, Berlin, Germany
4 JARA-Institute Molecular Neuroscience and Neuroimaging (INM-11), Forschungszentrum Jülich, Jülich, Germany
5 Child Neuropsychology Section, Department of Child and Adolescent Psychiatry, Psychosomatics and Psychotherapy, Faculty of Medicine, RWTH Aachen University, Aachen, Germany
6 Clinics for Psychiatry and Psychotherapy, Clinics at the Theodor-Wenzel-Werk, Berlin, Germany
7 Department of Psychiatry & Psychotherapy, University of Lübeck, Lübeck, Germany
8 Department of Psychiatry and Psychotherapy, Medical University of Vienna, Vienna, Austria
9 Comprehensive Center for Clinical Neurosciences and Mental Health, Medical University of Vienna, Vienna, Austria
10 Department of Clinical Psychology and Psychotherapy, Institute of Psychology, Friedrich-Alexander-Universität Erlangen-Nürnberg, Erlangen, Germany

Präambel:
Im Folgenden wollen wir versuchen, verschiedene in der Literatur gebräuchliche Begriffe zu definieren. Es gibt durchaus Überlappungen und Begriffe sind auch Teilmengen von Oberbegriffen. Sowohl die Definitionen als auch die gesetzlichen Anforderungen im Bereich der digitalen Gesundheitsversorgung sind hoch dynamisch. Digitale Gesundheitsanwendungen werden sich hoffentlich in Zukunft in der Versorgung etablieren.

IMI (Internet- und mobilbasierte Interventionen)

Internet- und mobilbasierte Interventionen (IMI) sind technologie-basierte, digitale Anwendungen, die über Computer oder mobile Endgeräte bereitgestellt werden und zur Unterstützung von Selbsthilfe, Selbstmanagement, (Selbst-)Monitoring und der Behandlung oder Prävention von Erkrankungen eingesetzt werden. Die Nationale Versorgungsleitlinie (NVL) Unipolare Depression führt den Begriff 2022 in der aktuellen Version ein und definiert IMI nur als evidenzbasierte digitale Interventionen, die psychoedukative Inhalte, therapeutische Module und gegebenenfalls Monitoring-Elemente enthalten. Anwendungen, die ausschließlich informativ-psychoedukativ sind oder lediglich Peer-Support durch Chats und Foren ermöglichen, werden explizit nicht als IMI betrachtet. IMI können sowohl als eigenständige Therapie als auch ergänzend zu konventionellen Behandlungen wie Psychotherapie oder Psychopharmakotherapie eingesetzt werden. Entscheidend für die Klassifikation als IMI ist die wissenschaftlich nachgewiesene Wirksamkeit. In Deutschland

zählen dauerhaft gelistete Digitale Gesundheitsanwendungen (DiGA) zu IMIs, da sie ihre klinische Evidenz (zumeist umgesetzt in einer randomisierten kontrollierten Studie (RCT)) nachweisen mussten.

DTx (Digital Therapeutics – Digitale Therapeutika)
Digitale Therapeutika (DTx) sind evidenzbasierte digitale Interventionen, die als Medizinprodukte reguliert und zur Prävention, Behandlung oder zum Management von Krankheiten eingesetzt werden. DTx müssen wissenschaftlich validierte therapeutische Effekte nachweisen und unterliegen regulatorischen Prüfungen, um ihre Wirksamkeit, Sicherheit und Qualität zu gewährleisten. Je nach Land sind diese Anforderungen unterschiedlich. In den USA werden DTx durch die U.S. Food and Drug Administration (FDA) als “prescription digital therapeutics” geprüft und zugelassen. In Deutschland fallen DTx unter das Digitale-Versorgung-Gesetz (DVG) und sind als DiGA reguliert.
Digitale Therapeutika (DTx) lassen sich in drei Kategorien einteilen: frei erhältliche DTx zur Unterstützung des Selbstmanagements, verschreibungspflichtige “prescription DTx” mit nachgewiesener klinischer Wirksamkeit, sowie “Combination DTx”, bei denen DTx ergänzend zu Medikamenten eingesetzt werden, um Behandlungsergebnisse zu verbessern.

DiGA (Digitale Gesundheitsanwendungen)
Digitale Gesundheitsanwendungen (DiGA) sind eine in Deutschland gesetzlich definierte Form digitaler Therapeutika, die vom Bundesinstitut für Arzneimittel und Medizinprodukte (BfArM) geprüft und zugelassen werden. Sie sind gemäß § 139e SGB V als erstattungsfähige digitale Medizinprodukte reguliert und können von Ärzt:innen oder Psychotherapeut:innen verordnet werden. DiGA dienen der Unterstützung von Diagnostik, Therapie oder dem Selbstmanagement von Erkrankungen und sind auch über das Fast-Track-Zulassungsverfahren als digitale Gesundheitsanwendung erstattungsfähig.

DiGA müssen hohe Anforderungen an Sicherheit, Datenschutz, Datensicherheit und Interoperabilität erfüllen. Zudem wird zwischen zwei Kategorien unterschieden:

- **Dauerhaft gelistete DiGA**: Diese haben ihre Wirksamkeit oder den Nutzennachweis (zumeist in einer randomisierten kontrollierten Studie (RCT)) von Beginn an oder im Zeitraum einer vorläufigen Listung nachgewiesen und erfüllen damit die evidenzbasierten Anforderungen des BfArM an eine digitale Gesundheitsanwendung. Sie sind uneingeschränkt erstattungsfähig und gelten als evidenzbasierte internet- und mobil-basierte Interventionen (IMI).
- **Vorläufige gelistete DiGA**: Diese haben noch keinen endgültigen Evidenznachweis erbracht und die vorläufige Zulassung basiert auf vorläufigen Pilotdaten oder einer systematischen Datenauswertung. Sie erhalten eine vorläufige Listung für zwölf bis maximal 24 Monate, während in dieser Zeit der klinische Nutzennachweis gemäß Anforderungskatalog des BfARm durchgeführt wird (bisher zumeist als randomisiert-kontrollierte Studie RCT umgesetzt). Falls die Evidenz nicht ausreichend belegt werden kann, wird die DiGA aus dem Verzeichnis entfernt.

DiGA sind gesetzlich als Teil des deutschen Gesundheitssystems verankert und unterscheiden sich damit von anderen digitalen Gesundheitslösungen. Sie müssen CE-zertifizierte Medizinprodukte der Risikoklasse I, IIa oder IIb sein und erhalten eine Pharmazentralnummer (PZN), die ihre Verordnung als Rezept und Abrechnung über die gesetzlichen Krankenkassen über 90 Tage ermöglicht.

Digitale Interventionen im Rahmen von Selektivverträgen (§ 140a SGB V)
Digitale Interventionen können auch im Rahmen von Selektivverträgen zwischen Krankenkassen und einzelnen Leistungserbringern erstattet werden. Solche Verträge ermöglichen es, digitale Versorgungsangebote gezielt für bestimmte Versichertengruppen bereitzustellen. Im Unterschied zu DiGA unterliegen diese Interventionen keiner zentralen Zulassung durch das BfArM. Die Qualitätssicherung erfolgt durch die Vertragsparteien, wobei gesetzlich vorgegeben ist, dass die allgemeinen Standards der Versorgung nicht unterschritten werden dürfen.

Ein Wirksamkeitsnachweis ist nicht erforderlich; die Krankenkassen können jedoch freiwillig Evaluations- oder Studienanforderungen in den Vertrag aufnehmen. Auch das Wirtschaftlichkeitsgebot wurde gelockert, sodass digitale Innovationen unter kontrollierten Bedingungen in der Versorgung eingesetzt werden können. Voraussetzung bleibt, dass Datenschutz und medizinische Sorgfalt gewährleistet sind, meist erfolgt dies über die Anforderung der Zertifizierung als Medizinprodukt und die Datenschutzrechtlichen und technischen Anforderungen an Software, um die Sicherheit zu gewährleisten.

Die Bandbreite der eingesetzten digitalen Interventionen reicht dabei von niedrigschwelligen Präventions-Apps bis hin zu komplexen digitalen und hybriden Versorgungsmodellen mit ärztlicher oder psychotherapeutischer Einbindung. Auch Medizinprodukte höherer Risikoklassen, etwa der Klasse IIa oder IIb, können – sofern entsprechend eingebunden – können Teil solcher Selektivverträge sein.

Präventions-Apps nach § 20 SGB V
Präventions-Apps sind digitale Angebote zur Gesundheitsförderung und Primärprävention, die von den gesetzlichen Krankenkassen gemäß § 20 SGB V gefördert werden dürfen. Dabei handelt es sich etwa um internetbasierte Programme, mobile Anwendungen oder hybride Formate, in denen klassische Kursinhalte (z. B. Edukation oder Übungsanleitungen) durch digitale Technologie vermittelt werden. Solche Apps richten sich an gesunde oder risiko-exponierte Personen und sind von den Digitalen Gesundheitsanwendungen (DiGA) nach § 33a SGB V abzugrenzen. Statt einer Verordnung durch Ärzt:innen oder Psychotherapeut:innen erfolgt die Nutzung in Eigeninitiative der Versicherten im Rahmen der Präventionsangebote ihrer Krankenkasse.
Um von den Kassen erstattet zu werden, müssen Präventions-Apps bestimmte Kriterien erfüllen und eine Zertifizierung durch die Zentrale Prüfstelle Prävention (ZPP) erhalten, die für drei Jahre gilt und bestätigt, dass diese den im Leitfaden Prävention des GKV-Spitzenverbandes definierten inhaltlichen und qualitativen Mindeststandards erfüllt. Anbieter

müssen ein fachlich fundiertes Konzept nachweisen (orientiert an anerkannten Präventionsprinzipien) und Qualitätskriterien wie wissenschaftliche Evidenz, Datenschutz und Nutzerfreundlichkeit erfüllen. Im Vergleich zu DiGAs sind die Anforderungen hier niedrigschwelliger.

Mobile Gesundheitstechnologien (mHealth)

Mobile Gesundheitstechnologien (mHealth) umfassen digitale Anwendungen, die über mobile Endgeräte wie Smartphones, Tablets oder Wearables bereitgestellt werden. Sie können zur Gesundheitsförderung, Prävention, Diagnostik oder Therapie eingesetzt werden. Der Begriff mHealth ist nicht gesetzlich definiert und umfasst eine breite Palette von digitalen Gesundheitsanwendungen.

DiGA sind eine spezifische Unterkategorie von mHealth, da sie als mobile Applikationen verfügbar sind und von Patient:innen ortsunabhängig genutzt werden können. Dennoch sind nicht alle mHealth-Anwendungen erstattungsfähig oder klinisch validiert. Fitness- und Lifestyle-Apps sowie viele präventive Gesundheits-Apps fallen nicht unter die Definition eines Medizinproduktes, da sie keinen medizinischen Zweck erfüllen.

Während mHealth-Anwendungen oft für gesundheitsbezogene Zwecke genutzt werden, erfüllen sie nicht zwangsläufig die regulatorischen Anforderungen, die an Medizinprodukte gestellt werden. In diesem Sinne sind mHealth-Anwendungen breiter gefasst und beinhalten sowohl nicht-regulierte als auch regulierte und zertifizierte digitale Gesundheitslösungen.
